# Utilization of an Integrated Medicare Advantage Model Is Positively Associated With Medication Adherence

**DOI:** 10.1093/geroni/igaa057.1550

**Published:** 2020-12-16

**Authors:** Renae Smith-Ray, Tanya Singh, Chester Robson

**Affiliations:** 1 Walgreens Center for Research on Health and Wellbeing, Deerfield, Illinois, United States; 2 Walgreen Co, Riverside, Illinois, United States; 3 Walgreen Co, Deerfield, Illinois, United States

## Abstract

An estimated 30% of U.S. healthcare costs are due to waste, inefficiencies, and excessive pricing. Research shows that integrated primary care models (IPC) improve health outcomes and reduce costs. Nearly all IPCs embed ancillary clinicians, including pharmacists, within the clinic. IPCs that embed a primary care clinic within a pharmacy are novel. This study describes the first known IPC for older adults that is based in a pharmacy and examines its impact on medication adherence. In January 2018, Walgreens launched an IPC focused on Medicare Advantage patients at select Kansas City Walgreens locations. Each morning the entire IPC team meets to review needs of patients who will be seen that day. Upon arrival, the patient is first seen by a pharmacist who completes medication and immunization reviews and fall risk screening. If a new medication is prescribed during the physician visit, the pharmacist returns to consult the patient. The IPC team works together to ensure that the Medicare Annual Wellness Exam is completed in entirety. We examined the impact of IPC utilization on adherence to the top seven chronic condition drug groups. IPC patients age 50+ with sub-optimal adherence (<80% proportion of days covered) during the year prior to the clinic opening were included (n=64). A Student’s t-test revealed an 11% improvement in optimal adherence year-over-year between the pre- and post- periods (p<0.001). The pharmacy-based IPC is associated with improved medication adherence. Future research should examine the impact of this model on patient satisfaction and additional health outcomes.

